# Comparative physiology reveals heat stress disrupts acid–base homeostasis independent of symbiotic state in the model cnidarian *Exaiptasia diaphana*

**DOI:** 10.1242/jeb.246222

**Published:** 2024-02-22

**Authors:** Luella R. Allen-Waller, Katelyn G. Jones, Marcelina P. Martynek, Kristen T. Brown, Katie L. Barott

**Affiliations:** Department of Biology, University of Pennsylvania, Philadelphia, PA 19104, USA

**Keywords:** Intracellular pH, Thermal stress, *Aiptasia*, Symbiosis, Climate change, Symbiodiniaceae

## Abstract

Climate change threatens the survival of symbiotic cnidarians by causing photosymbiosis breakdown in a process known as bleaching. Direct effects of temperature on cnidarian host physiology remain difficult to describe because heatwaves depress symbiont performance, leading to host stress and starvation. The symbiotic sea anemone *Exaiptasia diaphana* provides an opportune system to disentangle direct versus indirect heat effects on the host, as it can survive indefinitely without symbionts. We tested the hypothesis that heat directly impairs cnidarian physiology by comparing symbiotic and aposymbiotic individuals of two laboratory subpopulations of a commonly used clonal strain of *E. diaphana*, CC7. We exposed anemones to a range of temperatures (ambient, +2°C, +4°C and +6°C) for 15–18 days, then measured their symbiont population densities, autotrophic carbon assimilation and translocation, photosynthesis, respiration and host intracellular pH (pH_i_). Symbiotic anemones from the two subpopulations differed in size and symbiont density and exhibited distinct heat stress responses, highlighting the importance of acclimation to different laboratory conditions. Specifically, the cohort with higher initial symbiont densities experienced dose-dependent symbiont loss with increasing temperature and a corresponding decline in host photosynthate accumulation. In contrast, the cohort with lower initial symbiont densities did not lose symbionts or assimilate less photosynthate when heated, similar to the response of aposymbiotic anemones. However, anemone pH_i_ decreased at higher temperatures regardless of cohort, symbiont presence or photosynthate translocation, indicating that heat consistently disrupts cnidarian acid–base homeostasis independent of symbiotic status or mutualism breakdown. Thus, pH regulation may be a critical vulnerability for cnidarians in a changing climate.

## Teaser

**Summary:** Warming oceans threaten marine invertebrates. Heat disrupts acid–base homeostasis in a model symbiotic sea anemone regardless of symbiont presence or function, highlighting bleaching-independent effects of climate change.

## INTRODUCTION

The world's oceans have absorbed 93% of excess planetary heat from anthropogenic climate change (Johnson and Lyman, 2020), threatening the survival of sessile marine organisms that cannot migrate to cooler climates to avoid increasingly frequent and severe marine heatwaves (Atkins and Travis, 2010; Kim et al., 2023; Smith et al., 2023). Marine heatwaves have already decimated populations of keystone invertebrates, including notable losses of reef-building corals (scleractinians) (Hughes et al., 2017, 2018). Predicting how climate change will affect marine invertebrate populations is complicated by the fact that many species, including reef-building coral, engage in symbiotic relationships with microbial symbionts (e.g. dinoflagellates, fungi, bacteria), whose effect on host environmental response we are only beginning to understand (Apprill, 2020; McFall-Ngai et al., 2013). Symbiosis can facilitate physiological adaptation, as partner switching or ‘symbiont shuffling’ can provide mechanisms besides genetic evolution by which organisms can adapt to changing surroundings (Cunning et al., 2015b; Toby Kiers et al., 2010). Yet the interdependence between partners can also magnify the environmental sensitivities of mutualistic organisms, as both species must maintain function together under new conditions (Apprill, 2020; Bénard et al., 2020; Goulet and Goulet, 2021). Therefore, we need to be precise in our understanding of when an abiotic stressor such as elevated temperature is affecting a host, its symbiont(s), both partners, or even modifying the dynamics of the interaction itself.

Direct effects of abiotic stress on many symbiotic invertebrates remain uncharacterized because environmental change can also have indirect consequences by disturbing obligate symbiosis function. For example, endosymbiotic dinoflagellates (family Symbiodiniaceae) can meet up to 90% of coral energy requirements by providing glucose and other photosynthates (Burriesci et al., 2012; Davy et al., 2012; Falkowski et al., 1984; Muscatine et al., 1984). Yet this mutualism is highly temperature sensitive, as cnidarians lose their symbionts just 1–2°C above local summer mean temperatures in a process known as bleaching (Glynn, 1996; Hoegh-Guldberg et al., 2007). Although bleaching is a visually striking stress response and thus an indispensable environmental monitoring tool (Hughes et al., 2017), recent studies have shown that heat stress can perturb coral carbon metabolism (Innis et al., 2021; Rädecker et al., 2021) and calcification (Allen-Waller and Barott, 2023; Inoue et al., 2012) even when bleaching does not occur. A better understanding of these more cryptic cnidarian heat stress responses is essential for predicting coral responses to climate change, and may assist in discovering additional coral resilience biomarkers (Barshis et al., 2010) that will help to identify stress-tolerant corals that can survive warming oceans (Putnam et al., 2017; Van Oppen and Gates, 2006).

The consequences of heat stress for the metabolism and cell physiology of marine invertebrates remain underexplored (Melzner et al., 2022), and more physiological data are necessary to predict how accelerating climate change will alter species abundance and distribution (Wagner et al., 2023). For example, thermal stress interferes with intracellular pH (pH_i_) regulation in many species. This includes corals (Allen-Waller and Barott, 2023; Gibbin et al., 2015; Innis et al., 2021), for which pH regulation is essential not only to survival but also to calcification and thus reef persistence (Tresguerres et al., 2017). Understanding how heatwaves will affect cnidarian acid–base homeostasis is crucial as increasing atmospheric CO_2_ simultaneously acidifies and warms the oceans (Albright and Mason, 2013; Albright et al., 2016). However, the mechanism of thermal pH_i_ dysregulation in corals remains unclear. Disruption of symbiont CO_2_ uptake at high temperatures might lead to acidification in cells hosting endosymbionts (Gibbin et al., 2015); however, this does not explain why cells without symbionts are also more acidic after heat stress (Allen-Waller and Barott, 2023; Innis et al., 2021). ATP limitation resulting from symbiont loss might reduce available energy for acid–base homeostasis for the whole organism, as bleaching-susceptible individuals have lower pH_i_ than their bleaching-resistant neighbors during marine heatwaves (Innis et al., 2021). Yet even corals that do not bleach can suffer disrupted metabolism (Innis et al., 2021; Rädecker et al., 2021) and pH_i_ homeostasis (Innis et al., 2021) under heat stress. Symbiotic dysfunction is therefore unlikely to be the sole cause of thermal acid–base dysregulation. However, it remains unclear whether heat stress alters cnidarian pH_i_ by causing photophysiological stress in the resident Symbiodiniaceae, directly affecting host cellular processes, or by disrupting the mutualism itself.

To test the hypothesis that heat stress interferes with cnidarian acid–base regulation independent of symbiont loss, we conducted a comparative physiology analysis using symbiotic and aposymbiotic individuals from two cultures of a clonal line of the symbiotic sea anemone *Exaiptasia diaphana*, CC7. *Exaiptasia diaphana* is a robust model for cnidarian symbiosis that can live indefinitely in culture without symbionts (Sunagawa et al., 2009; Weis et al., 2008). This species thus allows us to disentangle the direct effects of heat stress on the host from stress resulting from bleaching, avoiding complications associated with the severe stress experienced by corals following symbiont loss at elevated temperatures (Brown, 1997; Davy et al., 2012; Jones, 2008; Oakley and Davy, 2018). In order to investigate host-derived changes in acid–base homeostasis in relation to physiological disruptions resulting from bleaching, symbiotic and aposymbiotic CC7 *E. diaphana* (Sunagawa et al., 2009) were exposed to four increasing temperature treatments for 2 weeks: 25°C (ambient control), 27°C, 29°C and 31°C. The experiment was repeated using two cohorts to test whether temperature responses were similar across populations acclimated to different laboratory culture conditions. We then measured symbiont density, protein content, pH_i_, symbiont and host carbon assimilation, and metabolic rates (photosynthesis and respiration). We hypothesized that elevated temperatures would decrease host pH_i_ in all anemones, but that higher rates of photosynthesis and photosynthate translocation to the host in anemones hosting more and/or more productive symbionts could mitigate pH_i_ dysregulation in symbiotic individuals. These data provide crucial insights into how climate change alters cellular homeostasis in the context of endosymbiosis.

## MATERIALS AND METHODS

### Anemone populations

**Fig. 1.**
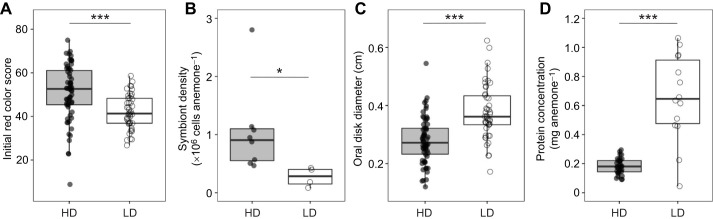
**Symbiotic individuals from two cohorts of clonal *Exaiptasia diaphana* showed baseline differences in symbiont density and body size.** (A) Pre-treatment color scores (% red color) of symbiotic anemones differed by cohort [high symbiont density (HD): *N*=61; low symbiont density (LD): *N*=45]. (B) Symbiont cells per anemone at baseline incubation temperature (25°C) differed by cohort (HD: *N*=8; LD: *N*=4). (C) Pre-treatment body size differed by cohort (HD: *N*=61; LD: *N*=45). (D) Protein concentration differed by cohort (HD: *N*=32; LD: *N*=14). Points represent individual anemones. Boxplots show medians with 25th and 75th percentiles, and whiskers show 1.5× interquartile range. Asterisks show results from linear mixed effects models (A,B,D) with cohort as fixed effect and anemone container as a random effect (*P<0.05, ***P<0.001), or linear regression with cohort as fixed effect (*P*<0.001).

Symbiotic and aposymbiotic *Exaiptasia diaphana* Rapp 1829 of the CC7 male clonal strain were used in this study (Sunagawa et al., 2009). Aposymbiotic and symbiotic animals from two distinct CC7 populations (subsequently termed cohorts) were used. One cohort had been maintained in the Barott Lab at the University of Pennsylvania for several years with static temperature (25°C) and salinity (35 ppt; filtered Instant Ocean artificial seawater), twice weekly feedings with freshly hatched *Artemia*, and photosynthetically active radiation (PAR) of 100 µmol m^−2^ s^−1^ on a 12 h:12 h light:dark schedule. The second cohort had been maintained in the Cleves Lab at the Carnegie Institution for Science for several years (25°C; 25 µmol m^−2^ s^−1^ 12 h:12 h light:dark; twice weekly *Artemia* feedings; 35 ppt salinity in filtered Instant Ocean artificial seawater). This second cohort was acclimated to all Barott Lab culture conditions for 3 weeks prior to the experiment. Cohorts were analyzed separately because symbiotic animals from the two cohorts differed in important characteristics. Specifically, the Barott Lab cohort anemones had less pigmentation and larger body sizes, and thus were termed low symbiont density (LD); the Cleves Lab cohort anemones were darker with smaller body sizes, and were termed high symbiont density (HD) (Fig. 1). In both cohorts, aposymbiotic animals were generated from a subset of the corresponding symbiotic population and were maintained in an aposymbiotic state for several years prior to the start of the experiments. Aposymbiotic animals were kept in the dark in opaque tubs to prevent symbiont re-colonization, and symbiotic anemones were kept in transparent tubs.

### Temperature treatment setup and monitoring

**Fig. 2.**
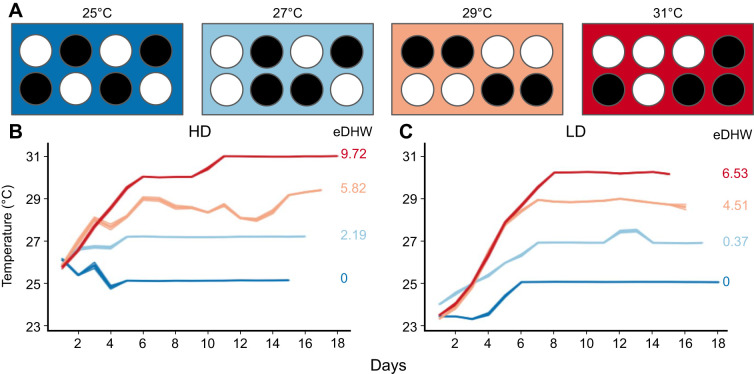
**Experimental design and temperature conditions across the experimental period.** (A) *Exaiptasia diaphana* were kept in separate containers (*N*=4 containers per treatment for each cohort; 3–4 anemones per container) within water baths held at 25°C, 27°C, 29°C or 31°C. Circles represent randomly arranged containers within each water bath (dark: aposymbiotic containers; white: symbiotic containers). (B,C) Daily average temperatures (24 h mean) across containers within temperature treatments of the (B) high symbiont density (HD) and (C) low symbiont density (LD) cohorts. Ribbons indicate standard error. Seawater temperatures were significantly different by treatment within each cohort (*P*<0.001). Annotations to the right of each temperature graph indicate total accumulated experimental degree heating weeks (eDHW; °C week^−1^) for each treatment.

Anemones were randomly assigned to each of the four temperature treatments for both experiments. Target temperatures (25°C, 27°C, 29°C and 31°C) were chosen to encompass a range of heat at and above anemone culture temperature (25°C) (Fig. 2). During the experiment, the anemones were kept in 118-ml containers (*N*=4 containers per treatment per symbiont status for each cohort, with 3–4 anemones per container; Fig. 2A) (Ziploc Twist ‘n Loc, SC Johnson, Racine, WI, USA) filled with 0.22 μmol l^−1^-filtered artificial seawater (FSW) (salinity ∼34 ppt; Instant Ocean Reef Crystals, Spectrum Brands, Blacksburg, VA, USA). Aposymbiotic and symbiotic anemones were placed in separate containers, and the exterior of all aposymbiotic containers and lids was covered with black electrical tape to maintain dark culture conditions. Containers with symbiotic anemones were covered with transparent plastic film secured with rubber bands to minimize evaporation while permitting light to pass through. All containers were arranged randomly on racks in water baths (40 gallons, ∼151.4 liters; one per temperature in each cohort) each equipped with a circulating pump (1500 l h^−1^ Submersible Water Pump, Vivosun, Ontario, Canada) and one to two 50 W heaters (either Aqueon Submersible Water Heater, Central Garden & Pet Company, Franklin, WI, USA, or Hygger Fish Tank Water Heater, Shenzhen Mago Trading Co., Shenzhen, China). Anemones were kept on a 12 h:12 h light:dark cycle (NICREW HyperReef LED, Shenzhen NiCai Technology Co., Shenzhen, China), with PAR ranging from 160 to 175 µmol m^−2^ s^−1^ for the duration of the experiment (Table 1). Water baths were arranged in a randomly assigned order for each separate experiment. Anemones were fed *Artemia* weekly through the experiments until 1 week prior to sampling, and anemone containers were cleaned every ∼3 days.

**Table 1.**
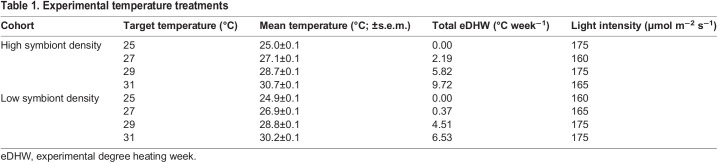
Experimental temperature treatments

Containers were placed inside of the tanks 2 days prior to each experiment to acclimate anemones to their new surroundings. All tanks started at 25°C on day 0 and were heated 1°C day^–1^ until they reached their target temperatures (4–8 days; Fig. 2B,C). Seawater temperatures were recorded every 15–120 s using cross-calibrated temperature loggers (accuracy: ±0.11 to 0.29°C; HOBO UA-001-64, Onset Computer Corporation, Bourne, MA, USA). Daily temperature measurements of the water bath were also recorded to ensure proper progression of the heat ramp (ProDSS with ODO/CT Probe, YSI, Yellow Springs, OH, USA). In addition, temperatures inside each anemone container were measured on six separate days through the experiment. As containers were consistently cooler than the surrounding water bath, we calculated each treatment's mean temperature (Fig. 2B,C) based on the mean difference (*T*_tank_–*T*_container_) subtracted from the water bath temperature. Anemones were monitored daily for mortality, and photographed every other day with a ruler and color standard at the bottom of each tub for size and color measurements. Anemone size and color were quantified in ImageJ (Abramoff et al., 2004). Size was measured by drawing a line across the widest point of each anemone's oral disk (excluding tentacles) and dividing the measured length by the measured length of a 1-cm line drawn on the ruler in the same image. Color was assessed using a modified coral color quantification protocol (Allen-Waller and Barott, 2023). Briefly, each image was converted to RGB, and the red channel was further examined. An ovoid region of interest was drawn to encompass each individual's oral disk, and intensity in that channel was divided by the intensity of the red standard in the same image to obtain percent intensity. Color scores are reported as [100%–(% red intensity)] because 100% red intensity corresponds to zero red pigment, a proxy for chlorophyll *a* concentration (Bahr et al., 2020).

Anemones within the same treatment were sampled on the same day (one treatment per day over a 4-day period) for the following metrics: one anemone from each container was measured for *in vivo* metabolic rates (HD cohort only), then frozen at −80°C for symbiont and protein content analysis; one was used for *in vivo* pH_i_ measurements; and one underwent a ^13^C stable isotope pulse-chase experiment. The order of sampling days differed between the HD and LD cohorts: the highest temperature was sampled on the first day in the LD cohort and on the last for the HD cohort.

### *In vivo* photosynthesis and respiration

Photosynthesis–irradiance curves were performed to determine photosynthetic and light-enhanced dark respiration (LEDR) rates for the HD cohort at the end of the 2-week experiment. Oxygen (O_2_) evolution was measured from 0 to 270 µmol m^−2^ s ^−1^ light on a subset of anemones (*n*=8 of each symbiotic status), totaling 32 anemones per treatment. Each anemone was individually enclosed in a 4 ml glass vial full of FSW and equipped with an optical O_2_-sensor (SV-PSt5, Presens, Regensburg, Germany). For each temperature treatment, four vials were filled with FSW and measured alongside anemones as blanks. Vials were then placed in a light-masked sensor dish reader (SDR SensorDish Reader with SDR-MSV24, Presens; factory pre-calibrated with Presens settings PSt5-2102-01_25°C, Presens SDR Version 4.0.0, https://www.presens.de/support-services/download-center/calibration-data) that optically measured O_2_ concentration in 24 vials simultaneously while being mixed continuously at 200 rpm on a plate shaker (Talboys Standard Orbital Shaker, Troemner, West Deptford Township, NJ, USA). Measurements were conducted inside of an incubator (B.O.D. Low Temperature Refrigerated Incubator, VWR, Radnor, PA, USA) set to each group's experimental temperature, and constant temperatures during incubation were verified using a temperature logger (accuracy:±0.11 to 0.29°C; HOBO UA-001-64, Onset Computer Corporation) placed in a water bath inside the incubator. O_2_ evolution was measured every 15 s for ≥10 min at each of six increasing light levels (24, 60, 113, 165, 217 and 270 µmol m^–2^ s^–1^), then in total darkness for ≥10 min. Each anemone's O_2_ evolution at each light level was quantified by plotting O_2_ versus time for the entire measurement period, manually subsetting the period where the slope was linear (5–10 min, starting ≥30 s after changing the light level to allow for light acclimation), and taking the slope of that linear period. Temperature- and light-specific blank values were determined by averaging O_2_ evolution across the FSW-only vials at each light level, and this value was then subtracted from each anemone's measured O_2_ evolution rate to account for microbial O_2_ evolution. A total of 12 individuals (1 symbiotic and 11 aposymbiotic) had O_2_ evolution rates within ±1 standard error of blank values in the dark, and were excluded from downstream analyses as they were deemed below the limit of detection, likely as a result of their small body size (55–140 µg protein per anemone). In total, *N*=15 symbiotic and *N*=5 aposymbiotic anemones were used for subsequent metabolic analyses. Total O_2_ released or consumed over time was determined from the concentration by multiplying by the volume of seawater in the vial (4 ml). All O_2_ evolution rates were then normalized to host protein content.

### Symbiont density and host protein content

Anemones were thawed and homogenized in 500 µl deionized H_2_O at 25,000 rpm for 10 s using a tissue homogenizer (Fisherbrand 850 Homogenizer, Thermo Fisher Scientific, Waltham, MA, USA) followed by needle-shearing until homogeneous using a 22-gauge needle. Tissue homogenates were spun at 7000 ***g*** for 5 min to separate host (supernatant) and symbiont (pellet) fractions. Symbiodiniaceae were resuspended in 1% sodium dodecyl sulfate in 1× phosphate-buffered saline, and cell concentrations were determined in triplicate using a flow-cytometer (Guava easyCyte 5HT, Luminex, Austin, TX, USA) as described previously (Innis et al., 2021; Krediet et al., 2015). Protein concentration in the host fraction was measured on a spectrophotometer (BioTek PowerWave XS2, Agilent, Santa Clara, CA, USA) using Coomassie Plus Bradford assay reagent (Pierce, Thermo Fisher Scientific).

### Intracellular pH

Cells were isolated from one randomly selected anemone from each anemone container (*n*=4 of each symbiotic state per treatment) by holding the anemone with forceps and brushing it against a small soft-bristled toothbrush submerged in 15 ml FSW for 2–4 min to break down tissue until enough isolated cells were obtained. The resulting cell suspension was passed through a 40 µmol l^−1^ cell strainer (Fisherbrand). Cells were then spun at 350 ***g*** for 4 min and resuspended in 1 ml FSW with 10 µmol l^−1^ pH-sensitive cell dye SNARF-1 acetoxymethyl ester acetate (Invitrogen, Thermo Fisher Scientific) and 0.1% dimethylsulfoxide (Invitrogen) as described elsewhere (Venn et al., 2009) for 20 min at 25°C in the dark. Live cells were then pelleted and resuspended in 1 ml FSW to remove any dye that did not enter the cells and imaged at 25°C in a glass-bottomed dish using an inverted confocal microscope (Leica SP8 DMi8, Leica Camera, Wetzlar, Germany) at 63× magnification (HC PL Apochromat C52 Oil objective, numerical aperture=1.4). All samples were excited at 514 nm (10% power argon laser, 1% emission, 458/514/561 beam splitter) and SNARF-1 fluorescent emission was simultaneously acquired in two channels (585±15 and 640±15 nm) using HyD detectors (gain=100, pinhole=1.00 airy unit). Using a scan speed of 400 Hz, 512×512 pixel images were acquired at 8 bits per pixel. Between 9 and 22 gastrodermal cells containing symbionts (symbiocytes) and 7 and 38 cells without symbionts (non-symbiocytes) were measured per symbiotic anemone; there were no symbiocytes present in aposymbiotic anemones, so only non-symbiocytes are reported. SNARF-1 fluorescence ratios in anemone cytoplasm were quantified using ImageJ and normalized to background fluorescence as described in Innis et al. (2021), and converted to pH values using a calibration curve generated on the same microscope within 2 weeks of the experiment as described in Venn et al. (2009) (Fig. S1).

### Stable isotope tracer experiment

One anemone per symbiotic anemone container (*n*=4 per treatment) was randomly selected to measure fixed carbon assimilation in the symbiont and translocation to the host using a stable isotope (NaH^13^CO_3_) pulse-chase experiment. In addition, three wild-type aposymbiotic anemones were included during the 25°C incubation as controls, expected to show no ^13^C assimilation owing to a lack of symbionts. At 11:00 h each sampling day, single anemones were placed in separate 50-ml conical tubes (Corning Falcon, Fisher Scientific) filled with FSW enriched with 31.22 µmol l^−1^ NaH^13^CO_3_ (Cambridge Isotope Laboratories, Andover, MA, USA). Anemones were then replaced in their experimental temperature and light conditions inside their sealed tubes, upside down in a tube rack submerged in their water bath, for a 7-h ‘pulse’ period so that symbionts could incorporate ^13^C via photosynthesis. After 7 h (18:00 h), the pulse was removed, anemones were rinsed with unamended FSW, and tubes were refilled with unamended FSW for the 12-h ‘chase’ period. At 06:00 h the following morning, the FSW was removed and anemones were frozen at −80°C and maintained at −80°C until processing. Samples were then thawed, homogenized and separated into host and symbiont fractions as described above. Each fraction was then transferred directly into a pre-weighed opened tin capsule (EA Consumables, Marlton, NJ, USA) and dried to stable weight for 24 h at 50°C. Capsules were then closed, weighed and shipped to the University of California, Santa Cruz Stable Isotope Facility (Santa Cruz, CA, USA) for ^13^C enrichment analysis by elemental analysis (NC2500 Elemental Analyzer, Carlo Erba Reagents GmbH, Emmendingen, Germany) coupled with isotope ratio mass spectrometry (Delta Plus XP iRMS, Thermo Scientific; coupling via: Conflo III, Thermo Scientific).

### Symbiont genotyping

Tissue slurries from 16 anemones from each cohort were used to verify Symbiodiniaceae identity at the species level. Symbionts were pelleted twice at 10,000 ***g*** for 5 min, and DNA was extracted using the DNeasy Plant Mini Kit according to the manufacturer’s instructions (Qiagen, Hilden, Germany). DNA quality was checked on a NanoDrop (Thermo Fisher Scientific) and three LD cohort samples were excluded from subsequent analysis owing to poor quality. PCR primers were chosen to distinguish between SSA01/*Symbiodinium linuche* (homologous symbiont) and SSB01/*Breviolum minutum* (heterologous symbiont present in other strain cultures in the Barott Lab). A fragment of the chloroplast 23S rDNA gene (cp23S) was amplified for two duplicate DNA samples from each anemone, one each using primers specific for SSA01 (F: 5′-CCTAATAACGACCTGCATGA-3′; R: 5′-TTTTGGTGATGATAAGCCGA-3′) and SSB01 (F: 5′-GACGGCTGTAACTATAACGG-3′; R: 5′-CCATCGTATTGAACCCAGC-3′) (Zhang et al., 2000) (Thermo Fisher Scientific). All reactions were performed using 0.2 µl Platinum Taq per polymerase reaction, Platinum Taq 10× PCR Buffer, 200 µmol l^−1^ dNTPs and 1.5 mmol l^−1^ MgCl_2_ (Thermo Fisher Scientific) with the following conditions: 94°C for 5 min; then 94°C for 30 s, 50°C for 1 min and 72°C for 2 min for 35 cycles; and finally 72°C for 10 min. PCR products were checked for amplification using a 1% agarose gel alongside no-DNA extraction and PCR controls (Table S1). Amplified products were purified using the Qiaquick PCR Purification Kit according to the manufacturer’s instructions (Qiagen). A subset were Sanger sequenced (ABI 96-capillary 3730XL Sequencer, Applied Biosystems, Waltham, MA, USA) and compared with all available Symbiodiniaceae cp23S sequences to verify that primers had amplified intended targets (either the SSA01 and SSB01 cp23S sequence, respectively) using Needleman-Wunsch Global Alignment in NCBI BLAST (Altschul et al., 1997). Purified products were checked on a NanoDrop and those with the highest yield were then digested using the HphI restriction enzyme in CutSmart 10× Buffer (New England Biolabs, Ipswich, MA, USA). Digests (39 µl each) and an undigested control were run on a 2% agarose gel to verify digestion at a common cut site (Table S1).

### Statistical analysis

All data were analyzed in RStudio version 2022.07.2 (https://www.rstudio.com/) and plots were generated using the package ggplot2 (Wickham, 2016). To find the best-fit model for each response variable, relevant linear, linear mixed effects, and generalized additive models were developed using the lme4 and mgcv packages (Bates et al., 2015; Wood, 2011). Corrected Akaike information criterion (AIC) values were calculated for each model using the MuMIn package (https://CRAN.R-project.org/package=MuMIn) and the model with the lowest AIC was chosen. Q–Q and residual plots were checked to ensure each model met normality and homogeneity of variance assumptions. All models are summarized in Table S2.

#### Temperature treatments

To compare temperatures of treatments within each cohort, one linear model analyzing daily average (24 h mean) container temperature with treatment as a fixed effect (four levels: 25°C, 27°C, 29°C and 31°C) was constructed for each cohort (HD and LD). To compare treatments between the two cohorts' experiments, a linear model analyzing daily mean container temperature with fixed effects of temperature (four levels: 25°C, 27°C, 29°C and 31°C) and cohort (two levels: HD and LD) was constructed. Significant interactions were further explored with Tukey's HSD adjusted *post hoc* pairwise comparison tests using the emmeans package (Lenth and Lenth, 2017). Experimental degree heating weeks (eDHW) were calculated for each treatment by summing the absolute value of the difference between the daily mean and 25°C, for all days over the course of the experiment where the daily mean exceeded 26°C (mean monthly maximum +1°C; Leggat et al., 2022). Mean monthly maximum was designated as 25°C because anemones were all maintained at 25°C before the experiment.

#### Cohort physiological comparison

The two experimental cohorts were compared for pigmentation (color) and size using data from both cohorts prior to temperature treatment. Cohort symbiont densities were assessed at the end of each experiment by comparing 25°C control anemones from each cohort. Temperature treatment was not found to have an effect on host protein, so protein densities from all temperatures were pooled within each cohort for between-cohort comparison. To verify that the pulsed ^13^C isotope was enriched in symbiotic anemones of both cohorts, carbon assimilation was compared between symbiotic host tissue, symbionts and aposymbiotic control host tissue using a linear model with effects of cohort (two levels: HD versus LD), symbiont status (two levels: symbiotic and aposymbiotic) and tissue fraction (two levels: host and symbiont).

#### Physiological response to temperature

Because cohorts differed in initial symbiont density and biomass, they were tested separately for the effect of temperature treatment (four levels: 25°C, 27°C, 29°C and 31°C) and symbiotic status (two levels: symbiotic and aposymbiotic) on anemone physiology (host protein, symbiont density, symbiont carbon assimilation, host carbon assimilation, symbiocyte pH_i_ and non-symbiocyte pH_i_). For each variable, the best model was selected according to the lowest AIC, after which any significant effects were further explored with Tukey's HSD adjusted *post hoc* pairwise comparison tests using the emmeans package (Lenth and Lenth, 2017). Notably, refined models of non-symbiocyte pH_i_ that did not consider symbiotic status all had lower AIC values than full models that accounted for symbiotic status, so aposymbiotic and symbiotic animals were combined for subsequent analysis of non-symbiocyte pH_i_.

#### Respirometry

To test effects of irradiance on O_2_ evolution, generalized additive models (GAMs) with light level as a fixed effect were fit separately to each photosynthesis–irradiance curve for each HD cohort symbiotic and aposymbiotic anemone from each temperature using a modified respirometry analysis procedure (Becker and Silbiger, 2020). The maximum oxygen evolution rate from each anemone's predicted photosynthesis–irradiance GAM fit was calculated and defined as that anemone's maximum estimated net photosynthetic rate (*P*_max_). The optimal temperatures for both *P*_max_ and light-enhanced dark respiration (LEDR) were then found by fitting two secondary GAM fits (one for *P*_max_ and one for LEDR) across all anemones, with experimental temperature as a fixed effect, and calculating the inflection points using the first derivative of that GAM spline. Best-fit GAMs for *P*_max_ and LEDR response to temperature were selected as described above.

#### Principal components analysis

For symbiotic animals only, differences in physiology were analyzed separately for each cohort using permutational multivariate analysis of variance (PERMANOVA) using the adonis function of the vegan package (Oksanen et al., 2013) and principal components analysis (PCA) using the R stats package prcomp function with temperature as a fixed effect. To assess differences between anemones with different symbiont densities, the subset of physiological response variables that were measured in all anemones (protein content, symbiont density and non-symbiocyte pH_i_) was used to compare all individuals using a separate PERMANOVA and PCA with cohort (two levels: HD versus LD) and symbiotic status (two levels: symbiotic versus aposymbiotic) as fixed effects.

## RESULTS

### Anemone cohort traits

Symbiotic anemones from the two experimental cohorts differed significantly in symbiont density (*F*=3.96, *P*=0.047) and color (*F*=13.86, *P*<0.001) at control temperatures (Fig. 1). Specifically, symbiotic anemones from one cohort were darker in color than the other (Fig. 1A) and hosted more symbionts per anemone (mean=1.05×10^6^ cells anemone^−1^; Fig. 1B); thus, they were termed high symbiont density (HD) anemones. Symbiotic anemones from the other cohort were paler (Fig. 1A) and contained fewer symbionts (termed low symbiont density or LD; mean=2.69×10^5^ cells anemone^−1^) (Fig. 1B). All anemones in which cp23S was successfully amplified contained a combination of SSA01 (*Symbiodinium linuche*) and SSB01 (*Breviolum minutum*) symbionts (Table S1).

Symbiotic LD anemones also contained more protein biomass per anemone than symbiotic HD anemones (*F*=75.94, *P*<0.001; Fig. 1D), corroborating observations of larger body size in the LD cohort (*F*=34.57, *P*<0.001; Fig. 1C). For both HD and LD cohorts, aposymbiotic anemones had significantly less protein biomass (HD: *F*=50.02, *P*<0.001; LD: *F*=57.71, *P*<0.001) and fewer symbionts (HD: *F*=229.2, *P*<0.001; LD: *F*=15.0, *P*<0.001) than their symbiotic counterparts (Fig. S2).

### Experimental conditions

Seawater temperatures in containers from all four treatments differed significantly from one another within both the HD (*F*=528.68, *P*<0.001; Tukey's HSD: *P*<0.001 for all pairwise comparisons) and LD (*F*=2682.3, *P*<0.001; Tukey's HSD: *P*<0.001 for all pairwise comparisons) cohort experiments. Between the two cohorts, the lowest three temperature treatments (25°C, 27°C and 29°C) did not differ (Tukey's HSD: *P*>0.15), whereas the highest temperature was an average of 0.5°C warmer in the HD cohort than in the LD cohort experiment (Tukey's HSD: *P*=0.017), reaching a maximum daily mean of 30.7°C and 30.2°C, respectively (Table 1, Fig. 2B,C). Total eDHW for the highest temperature treatments were 9.72°C week^−1^ for the HD and 6.53°C week^−1^ for the LD cohort (Table 1, Fig. 2B,C). Intermediate temperatures experienced eDHW ranging from 0.37 to 5.82°C week^−1^, and 25°C controls in both cohorts experienced 0°C week^−1^ (Table 1, Fig. 2B,C).

### Temperature effects on symbiont density and photosynthate assimilation

**Fig. 3.**
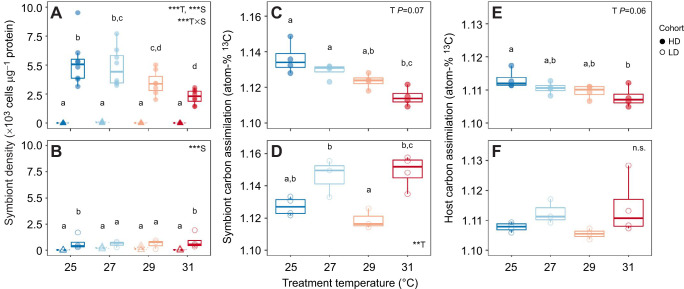
**Temperature altered dinoflagellate density and symbiotic function in high symbiont density but not low symbiont density *Exaiptasia diaphana*.** (A,B) Symbiont density response to temperature density in symbiotic (circles) and aposymbiotic (triangles) individuals differed between (A) high symbiont density (HD, *N*=8 per temperature) and (B) low symbiont density (LD, *N*=4 per temperature) anemone cohorts. (C–F) Temperature response of symbiont (C,D) and host (E,F) photosynthate assimilation (atom-% ^13^C) differed between (C,E) HD and (D,F) LD anemones (*N*=4 per temperature). Points represent individual anemones. Boxplots show medians with 25th and 75th percentiles, and whiskers show 1.5× interquartile range. Asterisks indicate results of linear models with effects of temperature (T), symbiotic status (S) and their interaction (**P<0.01, ***P<0.001). Different lowercase letters denote significant pairwise groupings (*P*<0.05) (Tukey's HSD).

Temperature differentially impacted symbiosis in the two anemone cohorts. As temperatures increased, HD anemones showed a significant decline in symbiont density (*F*=4.86, *P*=0.005; Fig. 3A; Fig. S2c) and a marginally insignificant trend of less symbiont photosynthate assimilation (*F*=3.00, *P*=0.072; Fig. 3C), which resulted in less host photosynthate assimilation at the highest temperature relative to 25°C controls (*T*=3.04, *P*=0.044; Fig. 3E). By contrast, elevated temperature did not cause significant reductions in symbiont density in LD anemones (*F*=0.06, *P*=0.98; Fig. 3B). Symbiont photosynthate assimilation in LD anemones was higher than in HD anemones (*T*=–4.39, *P*<0.001; Fig. S3a) and although LD symbiont photosynthate assimilation was significantly affected by temperature (*F*=9.49, *P*=0.003; Fig. 3D), higher temperatures did not consistently decrease LD host photosynthate assimilation (*F*=1.75, *P*=0.22; Fig. 3F). In fact, symbiont loss was only correlated with lower host photosynthate assimilation in HD anemones, and the correlation was weak (*F*=5.54, *P*=0.034, *R*^2^=0.23; Fig. S3b). In the LD cohort, anemones with fewer symbionts did not assimilate less photosynthate (*F*=0.12, *P*=0.73, *R*^2^=–0.07; Fig. S3c). Across both cohorts, aposymbiotic anemones assimilated significantly less ^13^C than symbiotic hosts (*F*=–2.11, *P*=0.040), which in turn contained significantly less ^13^C than their symbiont populations (*F*=–10.56, *P*<0.001), confirming that the ^13^C pulse successfully enriched photosynthates in symbiont and host fractions (Fig. S3a).

There was no effect of temperature on protein content in symbiotic or aposymbiotic anemones from either the HD (*F*=0.433, *P*=0.73) or LD cohort (*F*=0.312, *P*=0.816) (Fig. S2a,b). Symbiont density model results were consistent whether symbiont density was calculated as cells per animal or cells per microgram anemone protein (Fig. 3A,B; Fig. S3c,d), confirming that the observed changes in symbiont density per protein were not a result of changes in host biomass. Two symbiotic anemone containers (one container each from the 27°C and 29°C treatments; six anemones total) from the LD cohort experienced total mortality on day 14 and were not analyzed.

### Anemone metabolic performance

**Fig. 4.**
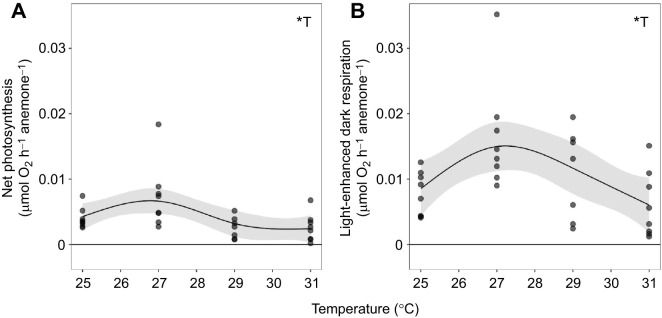
***Exaiptasia diaphana* metabolic rates vary with temperature.** (A) Estimated maximum net photosynthesis and (B) light-enhanced dark respiration of high symbiont density (HD) symbiotic anemones after incubation at treatment temperatures. Points in A represent generalized additive model-estimated maximum net photosynthetic rates for individual anemones and points in B represent actual measured dark oxygen evolution rates for individual anemones. *N*=8 anemones per temperature. Lines indicate metabolic rates predicted by generalized additive models for temperature (T) (gray ribbon, 95% confidence interval), with asterisks indicating significant effects (*P<0.05).

Photosynthesis–irradiance curves, measured only on the HD cohort of anemones, revealed significant metabolic changes across light levels in symbiotic anemones (edf: 1.98, *F*=70.77, *P*<0.001) and aposymbiotic anemones (edf: 1.94, *F*=11.83, *P*<0.001), with the highest photosynthesis and LEDR rates observed in the 27°C-incubated symbiotic group (Fig. S4a,b). Symbiotic anemones differed in their response to light from aposymbiotic anemones (symbiotic status×light: *F*=22.400, *P*<0.0001). Aposymbiotic anemones had lower O_2_ evolution, though it was still ≥0 at some light levels, indicating some photosynthetic activity (presumably owing to anemone surface-associated microbes) (Fig. S4a,b). Symbiotic and aposymbiotic groups were therefore treated separately for all subsequent respirometry analyses. GAMs fit to *P*_max_ and LEDR values for symbiotic individuals across the temperature treatments confirmed that temperature significantly affected both photosynthesis (edf: 2.79, *F*=4.22, *P*=0.021) and LEDR (edf: 2.33, *F*=4.46, *P*=0.030) in symbiotic anemones (Fig. 4). The predicted optimal temperature for *P*_max_ was 26.39±1.39°C (Fig. 4A), and the predicted temperature for maximum LEDR was 27.23±0.57°C (Fig. 4B). In aposymbiotic anemones, temperature significantly influenced LEDR (*F*=14.22, *P*=0.001) but not *P*_max_ (*F*=3.19, *P*=0.090) (Fig. S4c,d).

### Effect of elevated temperatures on anemone intracellular pH

**Fig. 5.**
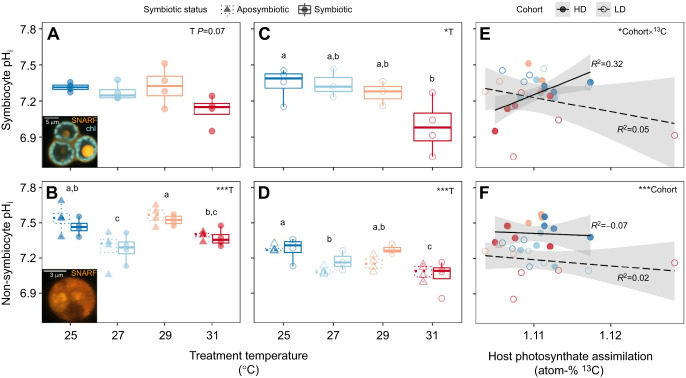
**Heat altered *Exaiptasia diaphana* intracellular pH (pHi_i_) regardless of symbiont presence, density or mutualistic function.** (A,B) pH_i_ of high symbiont density (HD) *E. diaphana* (A) symbiocytes and (B) non-symbiocytes varied with temperature. Insets: symbiocyte (A) and non-symbiocyte (B) showing fluorescence of pH-sensitive dye (SNARF-1) and symbiont chlorophyll (chl). (C,D) pH_i_ of low symbiont density (LD) *E. diaphana* (C) symbiocytes and (D) non-symbiocytes decreased with temperature. Asterisks beside inset T show results of linear models with temperature as an effect (*P<0.05, ***P<0.001); best-fit models found no effect of anemone symbiotic status in non-symbiocytes (symbiotic, circles; aposymbiotic, triangles). Different lowercase letters denote significant pairwise groupings (*P*<0.05) (Tukey's HSD). Points represent individual anemones. Boxplots show medians with 25th and 75th percentiles, and whiskers show 1.5× interquartile range. (E) In HD anemones, host photosynthate assimilation was correlated with symbiocyte pH_i_. In LD anemones, there was no correlation. (F) Host photosynthate assimilation did not affect non-symbiocyte pH_i_ in HD or LD anemones. Points show individual anemones. Regression lines show linear models with effects of host photosynthate assimilation (^13^C), cohort and their interaction; gray band represents 95% confidence interval; and asterisks show results of linear models (*P<0.05, ***P<0.001). Each point shows median pH of all cells from individual anemones (*N*=4 symbiotic+4 aposymbiotic anemones per temperature).

Temperature significantly affected pH_i_ of cells from both aposymbiotic and symbiotic anemones (Fig. 5). Symbiont-hosting cells (symbiocytes) tended to become more acidic with increasing temperature for both cohorts (Fig. 5A,C). This effect was significant in the LD cohort (*F*=4.25, *P*=0.035; Fig. 5C) but not significant in the HD cohort (*F*=3.00, *P*=0.073, Fig. 5A). Cells without symbionts (non-symbiocytes) showed a significant but non-monotonic temperature response (HD: *F*=10.97, *P*<0.001; LD: *F*=10.22, *P*<0.001), with 31°C-treated anemones having the most acidic cells in both cohorts (Fig. 5B,D). Within each cohort, there was no effect of anemone symbiotic state on pH_i_: aposymbiotic and symbiotic anemones had the same non-symbiocyte pH_i_ within each temperature, and showed the same thermal acid–base disruption (Fig. 5B,D). Interestingly, non-symbiocyte pH_i_ differed between cohorts, and was significantly higher in the HD cohort than in the LD cohort across temperatures (*F*=22.08, *P*<0.001; Fig. 5F). Moreover, host photosynthate assimilation only correlated with pH_i_ of symbiocytes from HD anemones (*F*=7.98, *P*=0.013, *R*^2^=0.32; Fig. 5E); there was no association between host photosynthate and pH_i_ of HD non-symbiocytes (*F*=0.056, *P*=0.82, *R*^2^=–0.07; Fig. 5F) or of either cell category in LD anemones (non-symbiocytes: *F*=0.76, *P*=0.40, *R*^2^=–0.02; symbiocytes: *F*=1.62, *P*=0.22, *R*^2^=0.05; Fig. 5E,F).

### Physiological separation by temperature and symbiotic status

**Fig. 6.**
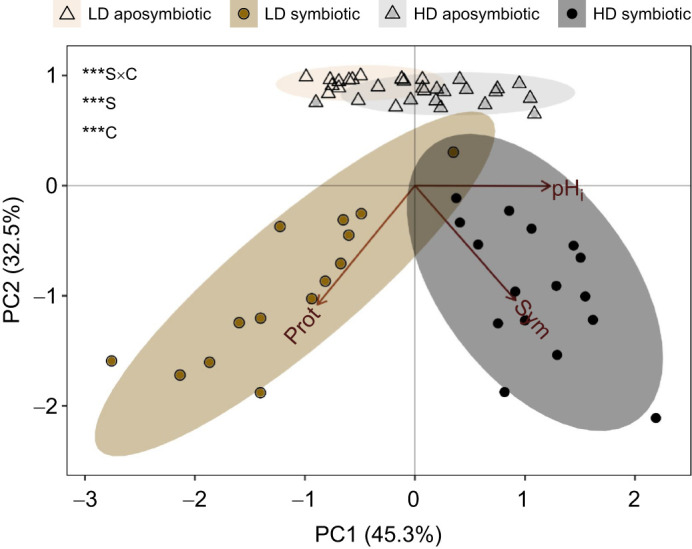
***Exaiptasia diaphana* physiology differed by degree of symbiont colonization.** Simplified principal components analysis for variables shared across all experimental replicates (Prot, protein; Sym, symbiont density; pHi, nonsymbiocyte intracellular pH) grouped by cohort and symbiotic status. Asterisks indicate significant PERMANOVA effects of cohort (C), symbiotic status (S) and their interaction (S×C) (***P<0.001). Ellipses show 95% confidence intervals for symbiotic status×cohort groupings. Points show individual anemones.

PCA revealed that symbiotic anemone physiology differed by temperature in both cohorts (HD: *F*=4.17, *P*=0.001; LD: *F*=2.12, *P*=0.026; Fig. S5). This consistent effect was largely driven by differences in pH_i_ and photosynthate assimilation (Fig. S5). However, separation of temperature groups differed between cohorts, largely because HD anemones in the hottest treatment assimilated less photosynthate than 25°C controls (Fig. S5a; Fig. 3E), whereas LD anemones under the same conditions assimilated more photosynthate than controls (Fig. S5b; Fig. 3E,F). Anemones also clustered separately by both cohort and symbiotic status. Consistent with their difference in overall temperature response, anemones from the two cohorts and symbiotic statuses all separated by host protein content and non-symbiocyte pH_i_, as well as by symbiont density (Fig. 6).

## DISCUSSION

### Elevated temperature causes intracellular acidosis regardless of organismal symbiotic status

Elevated temperatures consistently affected pH_i_ in aposymbiotic and symbiotic *E. diaphana*, tending to decrease nonsymbiocyte pH_i_ relative to animals at ambient temperatures. Furthermore, symbiotic anemone cohorts exhibited similar patterns of cellular acidification in response to increasing temperature despite differences in initial physiologies and bleaching responses. The consistency of these responses across different anemone populations indicates that neither endosymbiont presence nor their loss (i.e. bleaching) is necessary for acid–base dysregulation as temperatures increase above the thermal optimum of anemones. Further, the acidified pH_i_ we observed in the highest temperature treatments among all anemones (aposymbiotic, HD and LD) indicates that heat stress accumulated at this temperature in ways that assessments of symbiont density did not reveal. Although previous research in corals has suggested that heat stress alters pH regulation by interfering with symbiont photosynthesis and/or photosynthate translocation to the host (Allen-Waller and Barott, 2023; Cameron et al., 2022; Gibbin et al., 2015; Innis et al., 2021), the anemone responses recorded here indicate that heat can also impair cnidarian acid–base regulation independently of bleaching and the functional breakdown of the cnidarian–dinoflagellate symbiosis. These conclusions are not mutually exclusive, as heat stress likely disrupts both symbiont influence on and host control over host acid–base homeostasis. Multiple simultaneous host- and symbiont-derived pH regulatory mechanisms could help explain why heatwaves can impair coral acid–base homeostasis even in corals that do not lose their symbionts (Allen-Waller and Barott, 2023; Innis et al., 2021). Interestingly, bleaching resistance reduces the effect of heat on pH_i_ in corals (Innis et al., 2021), supporting contributions from both symbiont and host to acid–base dysregulation at high temperatures.

Many aquatic animals experience intracellular acidosis at high temperatures (e.g. Malan et al., 1976; Pörtner et al., 1999; Reeves and Malan, 1976). Some of this is likely driven by a passive physicochemical effect of temperature on intracellular buffer molecules, whereby bicarbonate, phosphate and protein dissociation constant (pK) values change with temperature (Van Dijk et al., 1997). Heat may also lead to cellular acidification by decreasing lysosomal membrane stability, causing H^+^ to leak from acidic lysosomes (Dimitriadis et al., 2012; Strand et al., 2017). These mechanisms may have contributed to lowered cellular pH_i_ observed across *E. diaphana* cell categories and symbiont densities at 31°C in the present study, and warrant further characterization in cnidarians. In addition, high temperatures increase metabolic demands in marine invertebrates (e.g. Brockington et al., 2001), including symbiotic cnidarians (Rädecker et al., 2021), and we hypothesize that this directly limits cellular capacity to regulate acid–base homeostasis. In support of this hypothesis, we observed significantly lower respiration and photosynthetic rates in symbiotic anemones held at our highest treatment temperature (31°C), suggesting metabolic impairment from accumulated heat stress that could substantially reduce available energy. Thermal energy limitations can reduce pH_i_ in marine invertebrates by reducing available ATP (Pörtner et al., 1999); in cnidarians, lower ATP levels would constrain the capacity of membrane-bound ATPases and related transporters (e.g. vacuolar H^+^-ATPase, Na^+^/K^+^-ATPase, Na^+^-H^+^ exchangers) that are essential for active pH regulation in different cellular compartments (Barott et al., 2015a,b, 2022; Tresguerres et al., 2017). Alternatively, cnidarians might respond to energy limitations by reducing expression of acid–base regulatory proteins (e.g. ion channels and transporters), as has been shown in reef-building corals (Bernardet et al., 2019; Kenkel et al., 2013). Further research is necessary to determine the relative contributions of these different possible mechanisms regulating cnidarian acid–base homeostasis.

### Bleaching response varied with initial symbiont density and body size

Anemone bleaching response varied with initial symbiont density, as anemones with high initial symbiont densities lost symbionts in the two highest temperature treatments, whereas anemones initially hosting lower symbiont densities did not. This result is consistent with greater bleaching sensitivity in cnidarians with denser symbiont loads (Cunning and Baker, 2013) and demonstrates the importance of considering variability of responses between different populations of the same clonal line. Although the HD cohort had to acclimate to higher light levels than in its previous culture conditions leading up to the experiment, this was controlled across temperature treatments, and the ∼50% reduction in symbiont density at 31°C relative to 25°C confirms this was a response to temperature and not to increased light intensity.

The two anemone cohorts also differed in their initial body size and protein biomass, which also may have influenced their bleaching responses. Fewer symbionts in larger-bodied LD anemones led to a higher host biomass:symbiont ratio, which could have prevented overloading of reactive oxygen species from symbiont dysfunction as temperatures increased (Cunning and Baker, 2013; Nesa and Hidaka, 2009; Wooldridge, 2009). Higher-biomass LD anemones may have also relied more on heterotrophy than the HD cohort (Hoogenboom et al., 2015), although both cohorts had equal access to food. In reef-building corals, higher heterotrophic feeding can compensate for symbiont loss (e.g. Grottoli et al., 2006) and can increase symbiont photosynthate assimilation (Krueger et al., 2018) and translocation from symbionts during heat stress (Tremblay et al., 2016). It is also possible that higher-biomass anemones had more energy reserves to catabolize in the absence of autotrophic resources during heat stress. Future studies should test the separate and combined influences of heterotrophy and symbiont density on *E. diaphana* bleaching susceptibility to better explain these observations.

Despite cohort differences in symbiont density and body size, symbiotic anemones from the lowest and highest temperatures separated in PCA consistently within each cohort, driven largely by changes in carbon assimilation and pH_i_. Differences in carbon assimilation across temperatures were expected, given that heat stress can decrease Symbiodiniaceae carbon assimilation (Ros et al., 2020) and translocation to cnidarian hosts (Allen-Waller and Barott, 2023; Baker et al., 2018; Tremblay et al., 2016). Yet our highest temperature treatment (31°C) only depressed host photosynthate assimilation in HD anemones with denser initial symbiont populations, even though the HD cohort assimilated less photosynthate overall relative to LD anemones at 31°C. The mechanisms driving this pattern are unknown, but it is possible that high temperatures increase HD symbiont competition over light, CO_2_ and/or nutrients (Cunning et al., 2015a, 2017; Hoogenboom et al., 2010; Wooldridge, 2009). Given the higher photosynthate assimilation of HD anemones than LD anemones under control conditions (25°C), along with their greater thermal sensitivity of carbon assimilation, it is also possible that HD anemones may have relied more on autotrophic energy than the LD cohort at permissive temperatures, rendering them more likely to bleach when temperatures increased. These cohort-dependent physiological differences within a clonal line of *E. diaphana* highlight the importance of repeating studies in different populations and accounting for cohort variation in this cnidarian model.

### Endosymbiosis modulates cellular response to heat stress

Symbiont presence within anemone cells modulated how host acid–base homeostasis was affected by heat stress: symbiocytes were most acidic at 31°C, whereas non-symbiocytes showed a dip in pH_i_ at 27°C relative to 25°C, and then decreased again at 31°C. The decline in pH_i_ of non-symbiocytes at 27°C corresponded with the peak metabolic rates of anemones, suggesting that metabolism may be important to these cells' non-linear response to temperature. Specifically, excess CO_2_ from high respiration rates at this temperature could acidify non-symbiocytes, but in symbiocytes, rapidly photosynthesizing symbionts at the same temperature may have mitigated this respiratory acidosis by drawing down dissolved inorganic carbon from the cytosol (Gibbin and Davy, 2014; Gibbin et al., 2014; Laurent et al., 2013; Putnam et al., 2017). Although all anemones in this experiment were dark acclimated for at least 40 min prior to imaging to reduce the effects of photosynthesis, it is 0.012w?>possible that anemones with more photosynthetically active symbionts had accumulated less CO_2_ prior to dark acclimation, leading to a higher cell pH_i_ that persisted even after dark acclimation.

Symbiocytes may also have differed from non-symbiocytes in their acidosis response due to differences in cytosolic buffering capacities between cell lineages, as is common in other species (Madshus, 1988). Because non-symbiocytes are defined simply as cells not containing Symbiodiniaceae, they could be of any host cell lineage, while symbiocytes are a single cell type originating from the gastrodermis (Glider et al., 1980). Non-symbiocytes and symbiocytes therefore likely have pH_i_ regulatory differences simply because cnidarian cell types differ greatly in cell contents and membrane-bound transporters (Levy et al., 2021). For example, dissolved inorganic carbon transport is a major factor affecting pH_i_ that is known to vary by cell type, as multiple coral species express Na^+^/K^+^-ATPase, sodium bicarbonate cotransporters and carbonic anhydrases with a high degree of tissue specificity (Barott et al., 2015b; Bertucci et al., 2011). Because acid–base homeostasis comprises diverse active and passive processes (Boron, 2004), we hypothesize cnidarian pH_i_ is governed by multiple different temperature-dependent mechanisms, a subset of which differ between cells with and without symbionts. Future studies should investigate cellular and molecular determinants of thermal pH_i_ dysregulation to determine which of these are specific to cells harboring intracellular symbionts.

Both cell category and initial organismal symbiont density affected the relationship between nutritional symbiosis function and pH regulation. Non-symbiocytes from the HD cohort consistently had higher pH_i_ than LD cohort non-symbiocytes. Interestingly, this pattern was independent of host photosynthate assimilation, and is therefore unlikely to result from our hypothesized trophic differences between cohorts. Instead, this persistent difference in pH_i_ setpoint once again suggests there are cohort-level differences in *E. diaphana* cellular physiology that warrant further exploration. In symbiocytes, although there was no overall relationship between host organic carbon assimilation and pH_i_, pH_i_ specifically increased with host photosynthate assimilation only in HD anemones. This could result from temperature sensitivity in symbiont inorganic carbon uptake; that is, if the low photosynthetic rates we observed under heat stress limited symbiont CO_2_ drawdown from the cytosol, high temperatures could simultaneously lower pH_i_ in symbiocytes relative to those at ambient temperatures while also decreasing the ability of symbionts to translocate photosynthate to the host. In this scenario, non-symbiocyte pH_i_ would have decreased at high temperatures for separate reasons unrelated to symbiosis. This is consistent with our reasoning that the host and symbiont play separate roles in regulating pH_i_.

Alternatively, heat stress could have decreased symbiont productivity by disrupting host acid-base regulation of the symbiosome. If pH_i_ dysregulation in symbiotic cnidarians does not depend on bleaching, then the inability to regulate pH at high temperatures could itself contribute to symbiosis breakdown. Loss of cnidarian host control over symbiosome contents has been linked to symbiotic breakdown (Cui et al., 2019; Rädecker et al., 2018, 2021; Xiang et al., 2020), and corals use a vacuolar H^+^-ATPase to concentrate inorganic carbon in the symbiosome to promote carbon fixation by the symbiont (Barott et al., 2015a). Cnidarian host pH_i_ dysregulation, whether by ATP limitation or changes to passive buffering, could therefore: (1) lead to accumulation of protons in the cytosol, decreasing symbiocyte pH_i_; and (2) contribute to the holobiont carbon limitation that is theorized to precipitate bleaching (Cunning et al., 2017; Rädecker et al., 2021; Wooldridge, 2009). This is consistent with our result that photosynthate assimilation decreased with low symbiocyte pH_i_ in the HD cohort, in which heat stress disrupted symbiotic function, but not in the putatively more heterotrophic LD cohort, which did not experience bleaching. Further research should test whether thermal acid–base dysregulation can initiate or exacerbate symbiont loss. Such a positive feedback mechanism between host and symbiont stress responses could lead to damaging synergistic effects as environmental change accelerates (Bénard et al., 2020).

### Conclusions

Here, we demonstrate that thermally induced acid–base dysregulation can occur independently of bleaching in cnidarians. Heat stress appears to impose metabolic constraints on *E. diaphana* that are separate from symbiont loss, which we hypothesize disrupt their ability to maintain acid–base homeostasis. This may have negative consequences for survival of cnidarians as well as other non-endosymbiotic marine invertebrates, as the ability to reallocate ATP to pH regulatory processes is thought to be a crucial mechanism of climate change resilience (Pan et al., 2015; Sokolova et al., 2012). Although endosymbiosis-specific effects on pH_i_ confirm that Symbiodiniaceae metabolism also contributes to host acid–base homeostasis, the direct effect of heat on host pH_i_ raises the intriguing possibility that thermal pH dysregulation precedes or even contributes to symbiont loss; however, additional study is necessary to validate this hypothesis. Regardless, as sea surface temperatures continue to rise (Johnson and Lyman, 2020) and marine heatwaves become more frequent and severe (Smith et al., 2023), it is crucial that we better understand how thermal stress impacts marine invertebrate cellular homeostasis. Future research on cnidarian thermotolerance should investigate specific mechanisms of ion transport disruption under heat stress. Ion transport is vital for fundamental processes including protein stability, endosymbiosis and calcification; its disruption, although less immediately visible than bleaching, is thus an existential threat to cnidarians (Tresguerres et al., 2014, 2017). Understanding thermal sensitivity of ion transport and acid–base homeostasis is particularly vital as increasing atmospheric CO_2_ simultaneously acidifies and warms the oceans (Albright and Mason, 2013; Albright et al., 2016; Harvey et al., 2013). Finally, our study also demonstrates how physiological experiments in model symbiotic organisms such as *E. diaphana* help address the broader question of how endosymbionts can mitigate and/or exacerbate host responses to rapid climate change, while highlighting that these effects are not uniform across populations.

## Supplementary Material

10.1242/jexbio.246222_sup1 Supplementary information
