# Supplementary material for: Comparative physiology reveals heat stress disrupts acid–base homeostasis independent of symbiotic state in the model cnidarian Exaiptasia diaphana
Source: J Exp Biol. 2024 Feb 22;227(4):jeb246222. doi: 10.1242/jeb.246222 (PMC10911193; doi:10.1242/jeb.246222)
Supplement: Supplementary information [file jexbio-227-246222-s1.pdf]

Supplementary Materials and Methods

Intracellular pH quantification

During imaging, cells were chosen for imaging based on two predetermined visual assessments: (1) sufficient SNARF-1 loading in the cell (i.e. strong yellow/orange fluorescence) and (2) perceived cellular health (i.e. lack of membrane blebbing). All suspensions were imaged between 30-90 minutes following that anemone's cell isolation, as the majority of cells in suspension were observed to die after this time.

All confocal images were analyzed in ImageJ (Abramoff et al. 2004). Cells contained in micrograph images were visually scored for membrane integrity based on their cellular smoothness, and only high-confidence healthy cells were selected for analysis. After selection, a region of interest was drawn within each coral cell's host cytoplasm that excluded any part of the acidic symbiosome or alga. The mean pixel intensity of this region was then quantified in the red (640 ± 15 nm) and yellow (585 ± 15 nm) channels. This process was repeated for each cell in the image. A background region in the same image was then selected to completely exclude any cells, and its mean pixel intensity was quantified in the red and yellow channels. Background mean pixel intensity in each channel was then subtracted from the mean pixel intensity in each channel of the cellular region(s) to obtain each cell's (i.e., cell 1 red channel intensity - background red channel intensity = cell 1 red fluorescence).

A calibration curve using cells of known pH was constructed to convert all cells' ratios of red and yellow fluorescence intensities to pH. Briefly, *E. diaphana* cells were isolated and incubated with SNARF-1AM in the same manner as for experimental animals (main text Methods). Cells were then spun down and resuspended in a calibration buffers of standardized pH and osmolarity (Venn et al. 2009) with 30µM nigericin (Invitrogen, Thermo Fisher Scientific), and incubated for 5-10 mins to permeabilize the membrane and allow them to equilibrate with the surrounding pH prior to imaging. At least 10 symbiocytes and 10 non-symbiocytes were imaged per pH point. This was repeated for six different pH values (pH 6, 6.5, 7, 7.5, 8, or 8.5). Fluorescence ratios were related to solution pH by linear regression (Fig. S1a). For experimental cells, fluorescence ratios were converted to pH using the following equation:

$$pH = pK_A - \log_{10}[(R-R_B)/(R_A-R)*(F_B/F_A)]$$

where R_A = calibration cells' 585nm/640nm fluorescence ratio at pH 8.5, R_B = calibration cells' 585nm/640nm fluorescence ratio at 6, F_B = calibration cells' 640nm fluorescence intensity at pH 8.5, F_A = calibration cells' 640nm fluorescence intensity at pH 6, and pK_A = x-intercept obtained from plotting the calibration curve's fluorescence logarithmic term against solution pH (Fig. 1b).

Journal of Experimental Biology • Supplementary information

REFERENCES

Abramoff, Magalhães, P. J. and Ram, S. J. (2004). Image processing with ImageJ. *Biophotonics Int.* **11**, 36–42.

Venn, A. A., Tambutté, E., Lotto, S., Zoccola, D., Allemand, D. and Tambutté, S. (2009). Imaging intracellular pH in a reef coral and symbiotic anemone. *Proc. Natl. Acad. Sci. U. S. A.* **106**, 16574–16579.

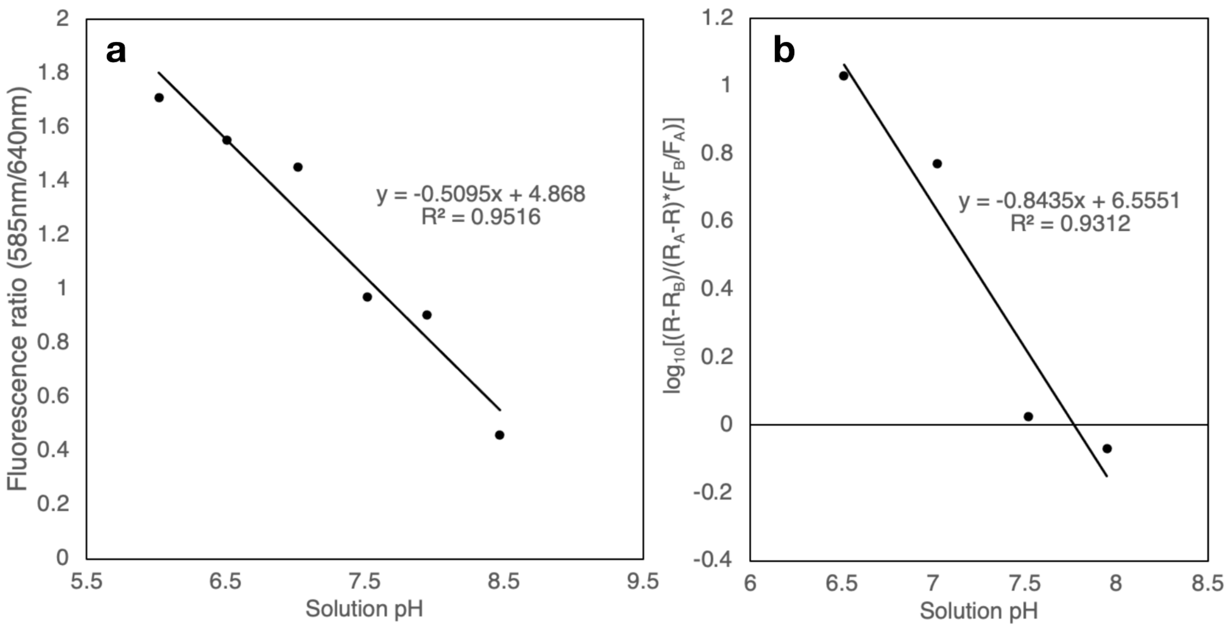

Fig. S1. *In vivo* calibration of the pH-sensitive dye SNARF1 in *Exaiptasia diaphana* cells. **a)** Inverse correlation between calibration solution pH and ratio (R) of SNARF1 fluorescence intensity at 585±15nm to 640±15nm. **b)** R was related to pH using the following equation: pH = $pK_A - \log_{10}[(R-R_B)/(R_A-R)*(F_B/F_A)]$ where R_A = 585nm/640nm fluorescence ratio at pH 8.5, R_B = 585nm/640nm fluorescence ratio at 6, F_B = 640nm fluorescence intensity at pH 8.5, F_A = 640nm fluorescence intensity at pH 6, and pK_A = x-intercept obtained from plotting the standard's logarithmic term against solution pH (shown). Each point represents N≥10 individual cells.

Journal of Experimental Biology • Supplementary information

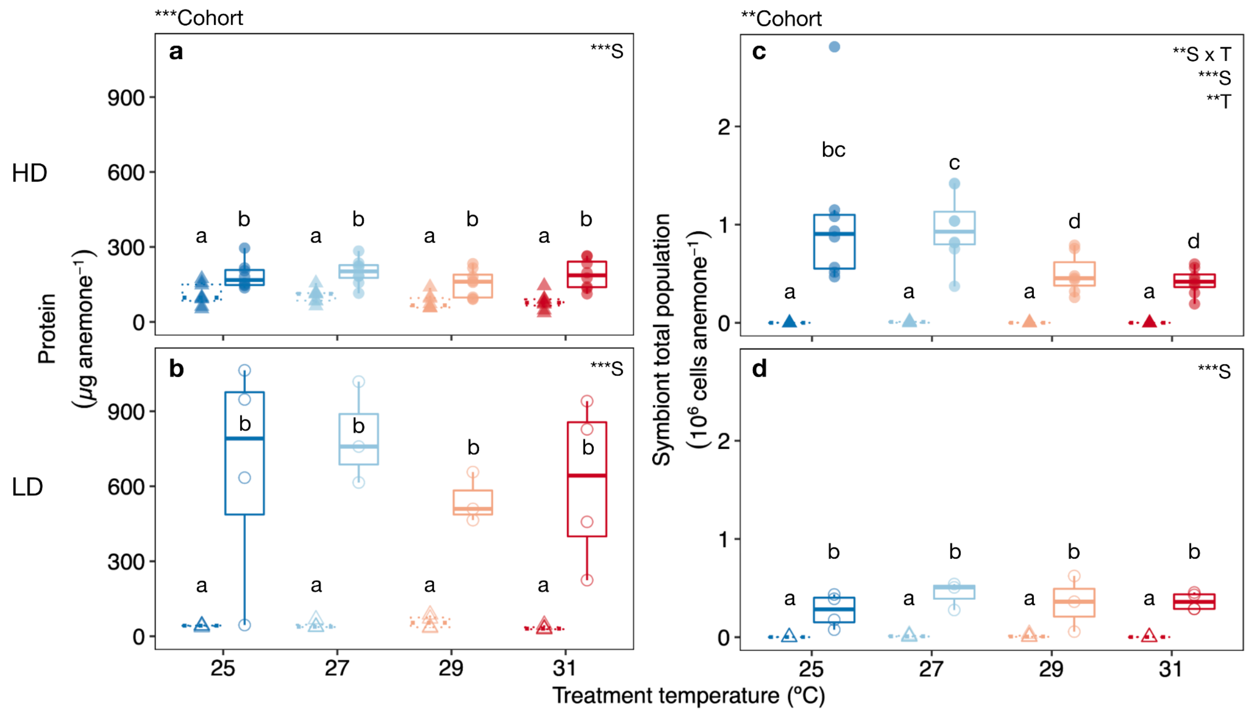

Fig. S2. Experimental cohorts differed in symbiotic *Exaiptasia diaphana* biomass and symbiont density. a-b) Total protein per animal in high-symbiont-density (HD) **(a)** and low-symbiont-density (LD) **(b)** cohorts. **c-d)** Total symbiont cells per animal in HD **(c)** and LD **(d)** cohorts. Superset annotations indicate results of linear models with effects of temperature (not significant) and cohort comparing symbiotic animals only. Inset capital letters show result of linear models with effects of temperature (T), symbiotic status (S), and their interactions (***p<0.001) within each cohort. Small letters denote significant pairwise groupings (p<0.05) (Tukey's HSD). Each point represents an average of 3 technical replicates from one individual anemones.

Journal of Experimental Biology • Supplementary information

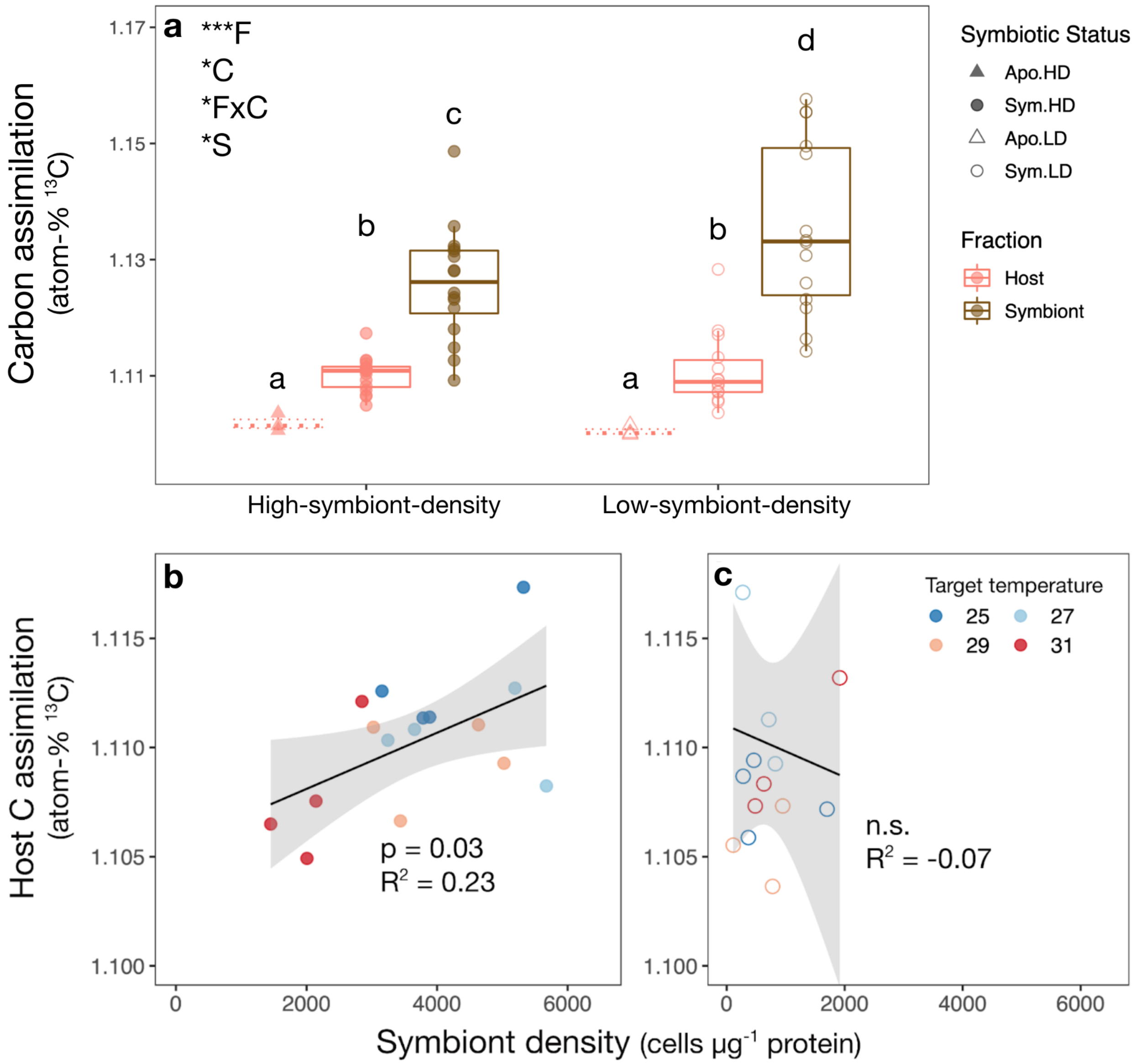

Fig. S3. ***Exaiptasia diaphana*** **photosynthate assimilation depended on initial symbiont density. (a)** ^{13}C isotope pulse-chase significantly enriched ^{13}C in symbiotic host (pink) and symbiont (brown) fractions from both high-symbiont-density (filled circles) and low-symbiont-density (open circles) *Exaiptasia diaphana* relative to aposymbiotic anemones (triangles). Inset capital letters show result of linear model with effects of fraction (F), cohort (C), symbiotic status (S), and their interactions (*p<0.05, ***p<0.001). Small letters denote significant pairwise groupings (p<0.05) (Tukey's HSD). **(b)** In the high-symbiont-density cohort, post-treatment host ^{13}C assimilation was positively correlated with symbiont density (linear model, df=1, f=5.54, p=0.03). **(c)** There was no relationship between host photosynthate assimilation and symbiont density in the low-symbiont-density cohort (linear model, df=1, f=0.12, p=0.73). Points represent individual anemones.

Journal of Experimental Biology • Supplementary information

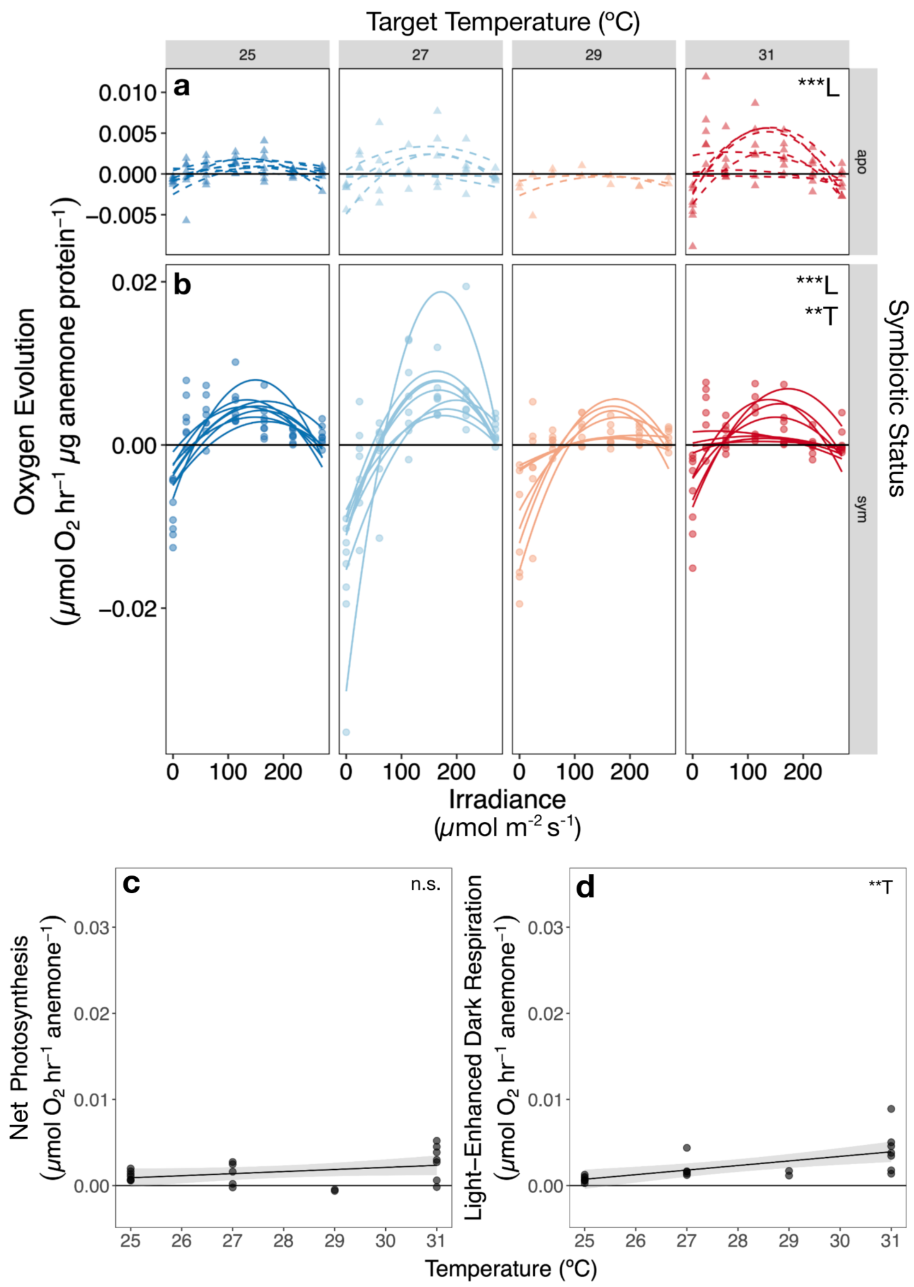

Fig. S4. Metabolic rates of aposymbiotic (a, c-d) and high-symbiont-density (b) *Exaiptasia diaphana* across treatment temperatures. Inset capital letters show effect of irradiance (L) on oxygen evolution in both aposymbiotic (**a**, triangles with dotted lines; edf=5.72, f=6.88, p<0.001) and symbiotic anemones (**b**, circles with solid lines; edf=5.90, f=40.2, p<0.001), but effect of temperature (T) only in symbiotic anemones (**b**; t=-2.849, p=0.005) (generalized additive models (GAMs) for irradiance with temperature as a factor, k=7). **c-d)** Estimated maximum net photosynthetic **(c)** and light-enhanced dark respiration **(d)** rates of aposymbiotic anemones across treatment temperatures. Points in **(a)** represent generalized additive model-estimated maximum net photosynthetic rates for individual anemones and points in **(b)** represent actual measured dark oxygen evolution rates for individual anemones. Lines show metabolic rates predicted by best-fit linear models for temperature (T) (gray ribbon = SE).

Journal of Experimental Biology • Supplementary information

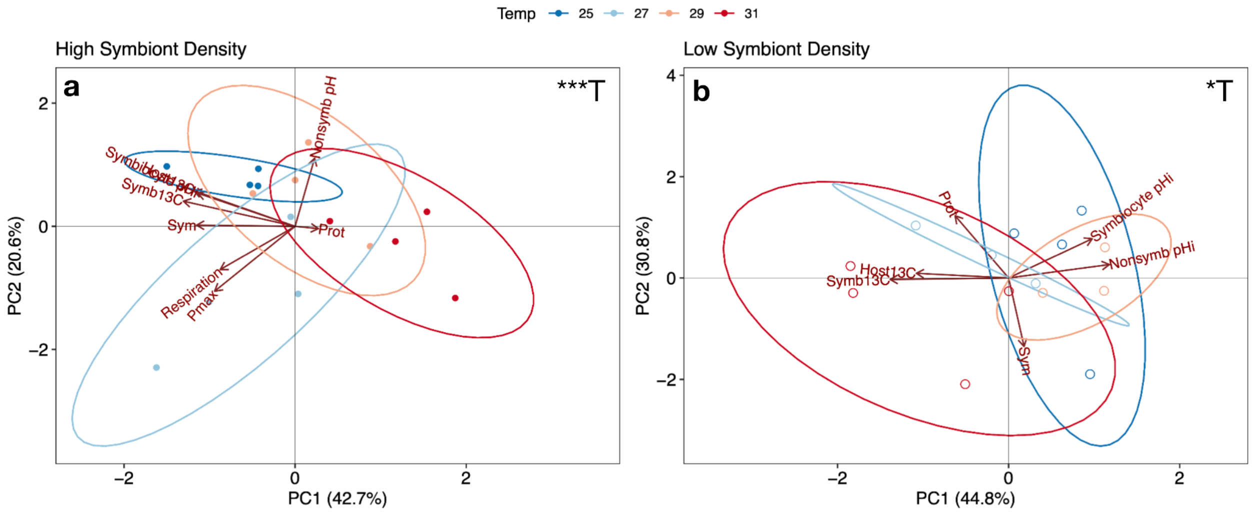

Fig. S5. Temperature altered physiology of symbiotic *Exaiptasia diaphana*. Principal components analysis with insets indicating effect of temperature (T) for **(a)** low-symbiont density (PERMANOVA, df=1, f=5.517, $p<0.001$) and **(b)** low symbiont density (df=1, f=2.487, p=0.06) anemones. Prot = protein; Sym = symbiont density; Symb13C = symbiont carbon assimilation; Host13C = host carbon assimilation; Symbiocyte pHi = symbiocyte intracellular pH; Nonsymb pHi = non-symbiocyte intracellular pH; Pmax = maximum net photosynthesis (HD only); Respiration = LEDR (HD only). Ellipses show 95% confidence intervals for temperature treatments. Points represent individual anemones.

Journal of Experimental Biology • Supplementary information

Table S1. Symbiodiniaceae genotyping results. Asterisk indicates the PCR product was used to confirm primer specificity by Sanger sequencing.

		**Primer set 1 (*S. linuche*)**		**Primer set 2 (*B. minutum*)**
**Cohort**	**Anemone**	**Amplification**	**Species confirmed by digest**	**Amplification**
HD	17	Y*	*S. linuche*	Y*
HD	18	Y	*S. linuche*	Y
HD	19	Y	*S. linuche*	Y
HD	20	Y	*S. linuche*	Y
HD	21	Y	*S. linuche*	Y
HD	22	N	N/A	N
HD	23	Y*	*S. linuche*	Y*
HD	24	Y	N/A	Y
HD	25	Y	N/A	Y
HD	26	Y	N/A	Y
HD	27	Y	*S. linuche*	Y
HD	28	Y	N/A	Y
HD	29	Y	N/A	Y
HD	30	Y	N/A	Y
HD	31	Y	N/A	Y
HD	32	N	N/A	N
LD	1	Y	*S. linuche*	Y
LD	2	Y	*S. linuche*	Y
LD	6	Y*	*S. linuche*	Y*
LD	8	Y	*S. linuche*	Y
LD	10	Y	*S. linuche*	Y
LD	13	Y	*S. linuche*	Y
LD	16	Y	*S. linuche*	Y
LD	18	Y	*S. linuche*	Y
LD	19	Y	*S. linuche*	Y
LD	20	Y	*S. linuche*	Y
LD	21	Y	*S. linuche*	Y
LD	30	Y	N/A	Y
LD	31	N	N/A	N

Table S2. Statistical models chosen by lowest AICc (*MuMIn* package).

	**Model**	**Response variable**	**Data subset**	**Fixed effects**	**Random effects**
**Temperature**	Linear model	Hourly temperature (°C)	HD cohort post-ramp	Temperature treatment	NA
	Linear model	Hourly temperature (°C)	LD cohort post-ramp	Temperature treatment	NA
	Linear model	Hourly temperature (°C)	Post-ramp	Temperature treatment, Cohort	NA
**Respirometry**	Linear model	Oxygen evolution (µmol O_2 min^{-1} L^{-1} $µg^{-1}$ protein)	N/A	Symbiotic Status, Light Level (µmol m^{-2} s^{-1})	NA
	Generalized additive model	Oxygen evolution (µmol O_2 min^{-1} L^{-1} $µg^{-1}$ protein)	Symbiotic anemones	Light Level (µmol m^{-2} s^{-1}), Temperature (°C)	NA
	Generalized additive model	Oxygen evolution (µmol O_2 min^{-1} L^{-1} $µg^{-1}$ protein)	Aposymbiotic anemones	Light Level (µmol m^{-2} s^{-1})	NA
	Generalized additive model	Estimated maximum photosynthesis (µmol O_2 min^{-1} L^{-1} µg protein)	Symbiotic anemones	Temperature (°C)	Anemone container
	Generalized additive model	Light-enhanced dark respiration (µmol O_2 min^{-1} L^{-1} µg protein)	Symbiotic anemones	Temperature (°C)	NA
	Linear regression	Estimated maximum photosynthesis (µmol O_2 min^{-1} L^{-1} µg protein)	Aposymbiotic anemones	Temperature (°C)	NA
	Linear regression	Light-enhanced dark respiration (µmol O_2 min^{-1} L^{-1} µg protein)	Aposymbiotic anemones	Temperature (°C)	NA
**Cohort baseline differences**	Linear mixed effects model	Red color score (% intensity)	Symbiotic anemones at the start of each experimental period	Cohort	Anemone container
	Linear mixed effects model	Symbiont density (cells $anemone^{-1}$)	Symbiotic anemones from	Cohort	Anemone container

Journal of Experimental Biology • Supplementary information

			25°C control groups		
	Linear mixed effects model	Protein (mg anemone^{-1})	Symbiotic anemones	Cohort	Anemone container
	Linear model	^{13}C assimilation (atom-% ^{13}C)	Symbiotic anemones + 6 wild type aposymbiotic controls	Cohort, Symbiont Status, Fraction	NA
	Linear model	Size (oral disk diameter)	Symbiotic anemones at the start of each experimental period	Cohort	NA
**Organismal physiology temperature response**	Linear model	Protein (µg anemone^{-1})	HD cohort	Temperature treatment, Symbiotic status	NA
	Linear mixed effects model	Symbiont density (cells µg protein^{-1})	HD cohort	Temperature treatment, Symbiotic status	Anemone container
	Linear mixed effects model	Symbiont density (cells anemone^{-1})	HD cohort	Temperature treatment, Symbiotic status	Anemone container
	Linear model	Symbiont ^{13}C assimilation(atom-% ^{13}C)	HD cohort	Temperature treatment	NA
	Linear model	Host 13C assimilation (atom-% ^{13}C)	HD cohort	Temperature treatment	NA
	Linear model	Protein (µg anemone^{-1})	LD cohort	Temperature treatment, Symbiotic status	NA
	Linear model	Symbiont density (cells µg protein^{-1})	LD cohort	Temperature treatment, Symbiotic status	NA
	Linear model	Symbiont density (cells anemone^{-1})	LD cohort	Symbiotic status	NA

	Linear model	Symbiont ^{13}C assimilation (atom-% ^{13}C)	LD cohort	Temperature treatment	NA
	Linear model	Host ^{13}C assimilation (atom-% ^{13}C)	LD cohort	Temperature treatment	NA
**Intracellular pH temperature response**	Linear model	Symbiocyte intracellular pH	HD cohort	Temperature	NA
	Linear model	Nonsymbiocyte intracellular pH	HD cohort	Temperature	NA
	Linear model	Symbiocyte intracellular pH	LD cohort	Temperature, Symbiont Status	NA
	Linear model	Nonsymbiocyte intracellular pH	LD cohort	Temperature	NA
	Linear regression	Symbiocyte pH_i	Symbiotic anemones	Host 13C assimilation (atom-% 13C), Cohort	NA
	Linear regression	Nonsymbiocyte pH_i	NA	Host 13C assimilation (atom-% 13C), Cohort	NA

Journal of Experimental Biology • Supplementary information
